# Clinical patterns and predictors of trauma-related mortality over 13 years: a retrospective analysis from a Level 1 National trauma center

**DOI:** 10.1186/s13017-025-00633-3

**Published:** 2025-07-05

**Authors:** Ayman El-Menyar, Sandro Rizoli, Ahammed Mekkodathil, Mohammad Asim, Sajid Atique, Abdel-Aziz Hammo, Hisham Jogol, Ahad Kanbar, Khalid Ahmed, Rafael Consunji, Husham Abdelrahman, Asmaa Al-Atey, Ahmad Kloub, Fernando Spencer Netto, Gustav Strandvik, Hassan Al-Thani

**Affiliations:** 1 Clinical Research, Trauma & Vascular Surgery, Hamad Medical Corporation, Doha, Qatar; 2 Clinical Medicine, Weill Cornell Medical College, Doha, Qatar; 3 Trauma Surgery Section, Hamad Medical Corporation, Doha, Qatar; 4 Department of Surgery, College of Medicine, Qatar University, Doha, Qatar

**Keywords:** Trauma, Mortality, Early death, Pre-hospital, In-hospital, Injury predictors

## Abstract

**Background:**

Qatar is one of six neighboring countries in the Gulf Cooperation Council region that form a political and economic alliance to foster multilateral cooperation. Given the shared challenges in trauma care, there is a need for a collaborative network to develop region-specific injury prevention strategies. For example, this study examines the clinical patterns and predictors of hospital mortality among trauma patients in Qatar.

**Methods:**

A retrospective analysis of trauma-related deaths (2010–2023) was conducted. Patients were stratified into early hospital mortality (EHM, ≤ 48 h) and late hospital mortality (LHM, > 48 h) groups. Further analyses examined in-hospital mortality (24 h, 24–48 h, 3–7 days, and > 7 days), age groups, injury mechanisms, and severity. A multivariable regression analysis identified predictors of early mortality.

**Results:**

Among 2,452 trauma-related deaths, 59% occurred in pre-hospital, while 41% occurred in-hospital. Compared to LHM (47%), EHM (53%) was associated with a younger age (35 vs. 39 years; *p* = 0.002), higher systolic blood pressure (0.82 vs. 0.67; *p* = 0.002), and diastolic blood pressure (2.03 vs. 1.75; *p* = 0.001). Motor vehicle crash (MVC) was the leading cause of death (35.3%), with vulnerable road users (VRU) the commonest in EHM (*p* = 0.004) and falls in LHM (*p* = 0.004). LHM was associated with a higher injury severity score (*p* = 0.001). On-admission systolic shock index independently predicted EHM (OR 2.23; 95% CI 1.09–4.52), while head (OR 7.14; 95% CI 2.44–20.00) and pelvic injuries (OR 3.70; 95% CI 1.19–11.11) and sepsis (OR 6.25; 95% CI 1.22–33.33) predicted LHM. In-hospital deaths exhibited a bimodal distribution, with peaks at 24 h (15%) and between the third and seventh days (10%). EHM showed an upward trend over the years (R² = 0.312), while LHM remained stable. Trauma-related mortality rates declined from 10.4 to 5.0 per 100,000 population (2011 and 2017) before rising to 9.7 by 2022. Pre-hospital deaths followed a similar pattern to the overall mortality, while the in-hospital rates remained steady. VRU-related injuries persisted at a high level, accounting for 26–43% of cases throughout the study period.

**Conclusion:**

This study highlights distinct trauma-related mortality patterns, with EHM linked to hemorrhage and shock, while LHM is associated with severe head injuries and sepsis. These findings underscore the need for targeted interventions to optimize bleeding control and address predictors such as shock indices for EHM and head injuries for LHM.

## Introduction

Traumatic injuries are a major global health challenge, contributing significantly to mortality, disability, and rising healthcare costs [1, 2]. Globally, an estimated 5.8 million deaths occur annually due to injuries, with road traffic accidents, self-inflicted injuries, and violence ranked as the three leading causes [3]. The widely recognized “golden hour” concept in trauma care underscores the critical importance of timely and effective interventions to mitigate early mortality following severe trauma [4]. This concept affirms that the first hour is decisive, and prompt care can significantly improve the patient’s outcomes. Advances in pre-hospital care, including rapid response systems and improved triage protocols, have enhanced survival rates by facilitating the timely delivery of life-saving interventions [5]. On the contrary, delays during this critical period may lead to irreversible complications, such as hemorrhagic shock, organ failure, or secondary brain injury, and worsening outcomes [6, 7].

Trauma-related mortality was traditionally described as following a trimodal distribution, with peaks corresponding to immediate, early, and late deaths post-injury [8]. However, recent studies propose a bimodal pattern attributed to reduced late mortality in advanced urban trauma systems [9]. This bimodal distribution consists of an initial peak of immediate deaths, followed by a secondary peak approximately 48 h after the injury [10]. Evidence suggests that the reduction of fatalities occurring one week after injury may explain this shift [5]. Yet, recent evidence has undermined these models, demonstrating heterogeneous mortality distributions without clear bi- or trimodal patterns [11].

Our previous report on trauma-related mortality patterns in the state of Qatar (a member of the Gulf Cooperation Council [GCC]) revealed that nearly half of the deaths (47.7%) occurred pre-hospital. In contrast, most in-hospital deaths occurred within 3–7 days (35%) [12]. Despite advancements in road safety, vehicle design, and trauma systems (e.g., pre-hospital care, faster transfers, and specialized trauma centers), immediate mortality rates persistently ranged between 30% and 60% [12–14]. These findings, in conjunction with evolving insights into mortality patterns, underscore the need for ongoing research to refine trauma systems, identify trends, and optimize preventive strategies. Therefore, this study examines the clinical patterns, trends, and predictors of trauma-related mortality, with emphasis on in-hospital deaths.

## Methods

We conducted a 13-year retrospective observational study to analyze all trauma-related deaths in Qatar between January 1, 2010, and June 30, 2023. This study utilized data from the Qatar National Trauma Registry (QNTR), a nationally representative registry maintained by the Hamad Trauma Center (HTC), Qatar. The QNTR is a national trauma registry that prospectively collects data on injury events, demographics, pre-hospital care, diagnoses, injury severity scores, in-hospital care, and outcomes of all trauma patients in Qatar, adhering to standards set by the National Trauma Data Bank (NTDB) and the Trauma Quality Improvement Program [TQIP] of the American College of Surgeons Committee on Trauma (ACS-COT). QNTR data undergo regular internal and external validation as part of TQIP [15, 16]. As the only tertiary trauma center in Qatar, HTC served as the sole site for this study. The study included patients who were admitted to the HTC following traumatic injuries and who died during hospitalization. Patients with incomplete time-to-death records and those who died at the scene were excluded from the analysis. We have also collected the number of pre-hospital deaths during the study period.

Data included patient demographics, injury mechanisms (such as traffic-related accidents, pedestrian injuries, falls from height, and other causes), and vital signs recorded upon admission to the emergency department (ED), including heart rate (HR), systolic blood pressure (SBP), diastolic blood pressure (DBP), Glasgow Coma Score (GCS) on admission, systolic and diastolic shock index (SI) and the injury characteristics using the injury severity score (ISS), Abbreviated Injury Scale (AIS). Complications such as ventilator-associated pneumonia (VAP), acute respiratory distress syndrome (ARDS), and sepsis were also documented. The primary outcome was early and late in-hospital mortality (EHM ≤ 48 vs. LHM > 48 h) and its predictors. Secondary outcomes included trends in pre-hospital and in-hospital mortality, as well as detailed in-hospital timing (categorized as first 24 h, 24–48 h, 3–7 days, and > 7 days), injury mechanisms, and severity. A population-based analysis of trauma-related deaths was conducted using QNTR data and population statistics from the National Planning Council, Qatar [17]. The systolic SI was calculated as HR divided by SBP, and the diastolic SI was calculated as HR divided by DBP. Vulnerable road users (VRUs), defined as pedestrians, roadway workers, wheelchair or personal mobility device users (motorized or non-motorized), electric scooter riders, bicyclists, and motorcyclists, were identified as high-risk due to their limited protection in traffic accidents [18].

### Ethical approval

for this study (Medical Research Center (MRC)# 12263/12 & 01/18/330 & 01/23/671) was granted by the Institutional Review Board of Hamad Medical Corporation, Doha, Qatar.

### Statistical analysis

Data were presented as proportions, medians (with interquartile ranges, IQR), or means (± standard deviations), as applicable. Patients were stratified into two groups based on the timing of death (EHM ≤ 48 h and late LHM > 48 h]), and comparative analyses were performed. Differences between EHM and LHM groups were assessed using the chi-square test for categorical variables and the student’s t-test for continuous variables. Subgroup analyses of pre-hospital and in-hospital mortality, stratified by age, injury mechanisms, and severity, were also conducted.

For older patients, mortality was analyzed using a cutoff age of 55 years, as described previously [19]. Additionally, age-based analysis was performed across seven 10-year intervals: 0–10, 11–20, 21–30, 31–40, 41–50, 51–60, and 61 years and older. The case fatality rate (CFR; defined as the ratio of in-hospital trauma-related deaths to total trauma admissions) and hospital mortality rates per 100,000 population were calculated using mid-year data from June 2010 to July 2023. To identify the predictors of early versus late in-hospital mortality, a multivariable logistic regression analysis was performed. Variables included in the model were those that showed significant differences in the univariate analysis, such as age, systolic shock index, ISS, GCS, sepsis, massive blood transfusion, and the presence of abdominal, head, and pelvic injuries. Results were expressed as adjusted odds ratios (aOR) with corresponding 95% confidence intervals (CI). A two-tailed p-value of < 0.05 was considered as a statistically significant difference. The statistical analyses were conducted using SPSS version 28 (IBM Corp., Armonk, NY, USA). Temporal trends in mortality (including pre-hospital, early in-hospital, and late in-hospital deaths) from 2011 to 2022 were analyzed by plotting the proportions of deaths over time. The coefficient of determination (R^2^) trendline was calculated to assess the strength of the trend. Temporal trend analysis was performed using Microsoft Excel (Microsoft Corp., Redmond, WA, USA).

## Results

### Early vs. late hospital mortality (EHM vs. LHM)

**Fig. 1:**
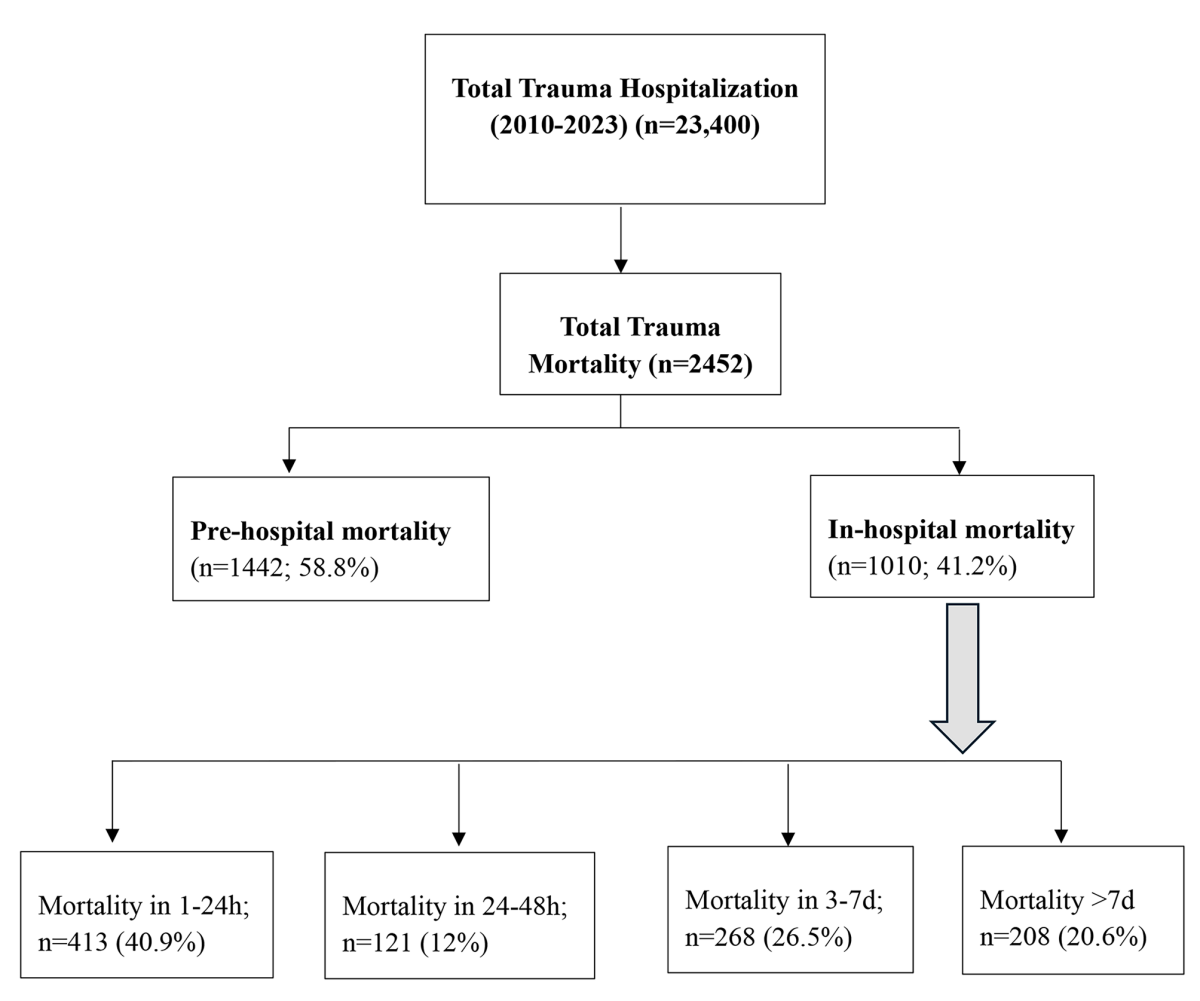
Time-based trauma-related mortality for the period from 2010 to 2023

**Fig. 2:**
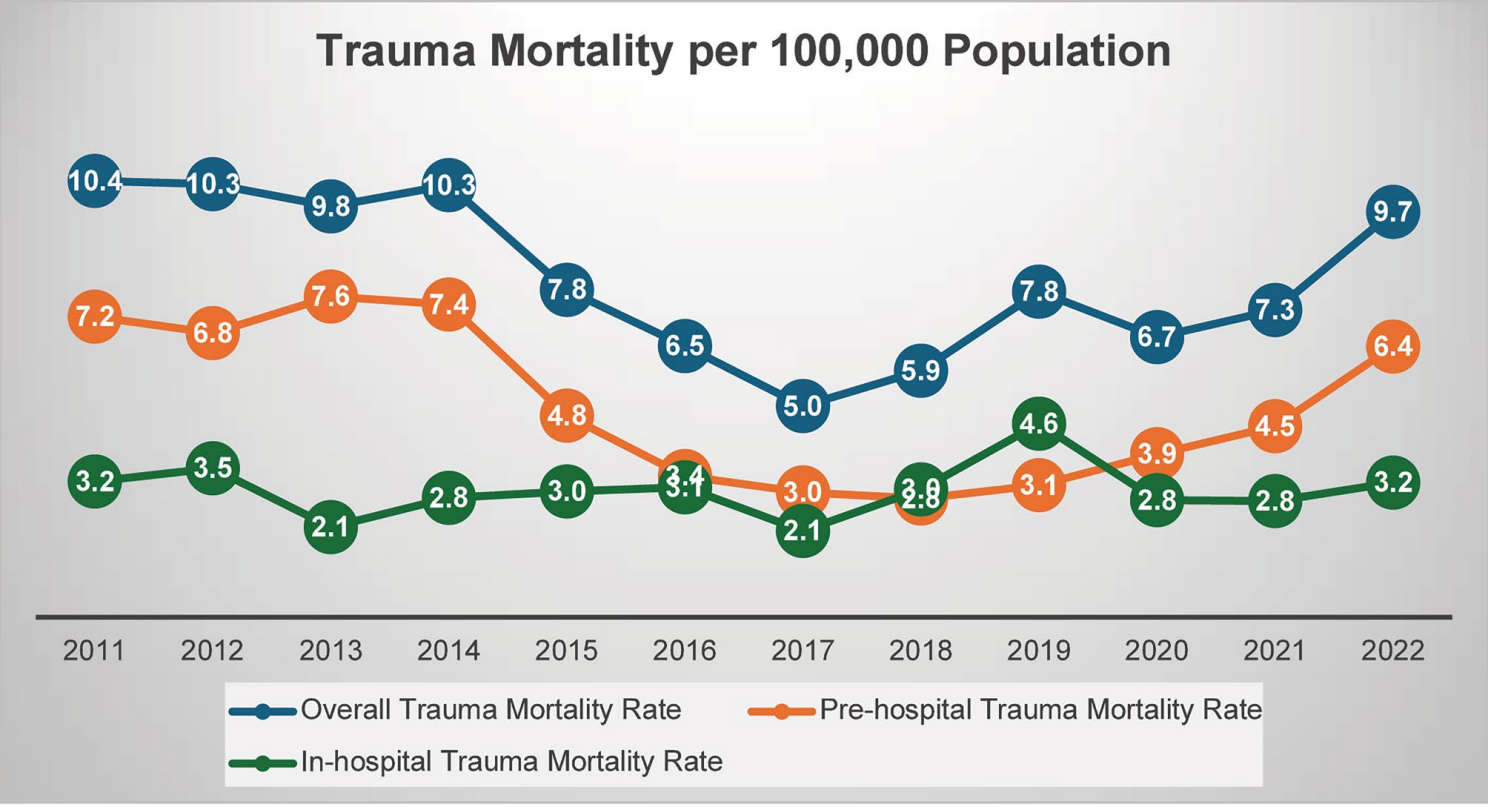
Overall (pre-hospital* and in-hospital) trauma mortality rates per 100,000 population in Qatar (2011–2022) (*the pre-hospital mortality data was not available as a full year in 2010 and in 2023)

Among the 22,618 trauma admissions, there were 2,452 trauma-related deaths, of which1,010 deaths occurred in the hospital (4.5% of the total admissions and 41% of trauma-related deaths) over the study period. Early mortality (EHM, ≤ 48 h) accounted for 53% of cases, while late deaths (LHM, > 48 h) comprised 47% of in-hospital deaths (Fig. 1). Most deaths (58.8%) occurred pre-hospital. The in-hospital case fatality rate (CFR) averaged 4.2% (range: 2.4-7.2%). Figure 2 demonstrates temporal changes in trauma-related mortality rates per 100,000 population in Qatar from 2011 to 2022. From 2011 to 2017, mortality rates declined, with overall mortality falling from 10.4 to 5.0 per 100,000 population in 2017 (R² = 0.26). Subsequently, mortality rates (both pre-and in-hospital) increased, reaching 9.7 per 100,000 population by 2022. Pre-hospital mortality followed a similar fluctuating trend, with a marked decrease until 2018, followed by a notable rise thereafter (R² = 0.29). In-hospital mortality remained relatively stable, declining from 3.2% in 2011 to 2.1% in 2017, before rising again to 3.2% in 2022, with a peak of 4.6 per 100,000 population in 2019 (R² = 0.01).

In-hospital fatalities (41.2%) were categorized into four time intervals. The highest proportion of in-hospital mortality occurred within the first 24 h (40.9%), followed by deaths between days 3–7 (26.5%). The mean age was 37.3 ± 18 years, with the early-mortality group being significantly younger (35 vs. 39 years, *p* = 0.002). Almost 17% of all mortalities occurred in patients aged > 55 years. Males predominated in both groups (93%).

Motor Vehicle Crashes (MVCs) were the leading cause of death (35.3%), with no significant difference between the EHM and LHM groups. However, VRUs were more frequent in the EHM group (32.8% vs. 27.7%, *p* = 0.004), whereas falls from height were more common in the LHM group (27.9% vs. 17.6%, *p* = 0.004) (Table 1). The median systolic SI was significantly higher in the EHM group (0.82 vs. 0.67, *p* = 0.002), with a larger proportion of patients in this group exhibiting a systolic SI ≥ 0.90 (71% vs. 52%, *p* = 0.001). Similarly, the median diastolic SI was elevated in the EHM group (2.03 vs. 1.75, *p* = 0.001), and a higher proportion had a diastolic SI greater than 2.2 (35% vs. 23%, *p* = 0.01).

**Table 1:** Demographics, injury mechanisms, clinical characteristics, management, and complications based on Time-to-Death of In-Hospital mortality

	Overall (*n* = 1010)	Early ≤ 48 h (*n* = 534; 52.9%)	Late > 48 h (*n* = 476; 47.1%)	*P* value
**Age (mean ± SD)**	37.3 ± 18.1	34.9 ± 16.7	39.1 ± 18.7	0.002
**Males**	934 (92.5%)	488 (91.4%)	446 (93.7%)	0.162
**Mechanism of injury**				
Motor Vehicle Crashes (MVC)	357 (35.3%)	193 (36.1%)	164 (34.5%)	0.004 for all
Vulnerable Road Users (VRU)	307 (30.4%)	175 (32.8%)	132 (27.7%)	
Falls	227 (22.5%)	94 (17.6%)	133 (27.9%)	
Violence-related injuries (VRI)	36 (3.6%)	21 (3.9%)	15 (3.2%)	
Hit by heavy objects	27 (2.7%)	17 (3.2%)	10 (2.1%)	
Others	56 (5.5%)	34 (6.4%)	22 (4.6%)	
**EMS transport time** min, (Median & IQR	70 (52–88)	68 (50–89)	71 (55–88)	0.26
**Injury severity score (IQR) (** ***n*** ** = 880)**	30 (25–38)	29 (20–38)	30 (26–38)	0.002
**ISS > 25**	673 (76.5%)	281 (69.0%)	392 (82.9%)	0.001
**GCS at ED (IQR)**	3 (3–3)	3 (3–3)	3 (3–8)	0.001
Systolic Shock index (SSI)	0.71(0.51–1.02)	0.82(0.59–1.10)	0.67(0.49–0.92)	0.002
SSI ≥ 0.90	362 (58.2%)	146 (70.9%)	216 (51.9%)	0.001
Diastolic Shock index (DSI)	1.84(0.66–2.23)	2.03(1.46–2.32)	1.75(0.57–2.26)	0.001
DSI ≥ 2.2	127 (26.5%)	45 (34.9%)	82 (23.4%)	0.01
**Ethanol positive**	28 (2.8%)	21 (3.0%)	19 (3.8%)	0.476
**Associated injuries**				
Head	691 (83.0%)	284 (74.0%)	407 (90.6%)	0.001
Chest	571 (73.0%)	280 (74.5%)	291 (71.7%)	0.379
Abdomen	317 (49.4%)	170 (57.8%)	147 (42.2%)	0.001
Pelvis	198 (31.3%)	107 (36.1%)	91 (27.1%)	0.014
**Intubation**	794 (98.9%)	402 (99.0%)	392 (98.7%)	0.712
**Exploratory laparotomy**	118 (29.5%)	64 (41.0%)	54 (22.1%)	0.001
**Craniotomy/ craniectomy**	74 (18.6%)	6 (4.4%)	68 (26.1%)	0.001
**Blood Transfusion**	629 (83.1%)	279 (83.0%)	350 (83.1%)	0.971
**Blood units transfused**	9.9 ± 10.4	10.6 ± 9.5	9.8 ± 11.1	0.229
**MTP activation**	226 (52.7%)	118 (68.2%)	108 (42.2%)	0.001
**VAP**	93 (16.5%)	6 (2.4%)	87 (27.9%)	0.001
**ARDS**	47 (8.5%)	4 (1.6%)	43 (14.3%)	0.001
**Sepsis**	56 (9.9%)	3 (1.2%)	53 (17.0%)	0.001
**Renal complications**	8 (4.2%)	1 (0.9%)	7 (9.3%)	0.005

Median, IQR: Interquartile range; ED: emergency department; VAP: ventilator-associated pneumonia; ARDS: acute respiratory distress syndrome;

Head injuries were more frequent in the LHM group (91% vs. 74%, *p* = 0.001), with higher AIS scores for the head (4.6 vs. 4.3, *p* = 0.001). In contrast, EHM was significantly associated with hemorrhagic injuries, particularly abdominal trauma (58% vs. 42%, *p* = 0.001), as reflected by elevated AIS scores for both the abdomen (3.1 vs. 2.7, *p* = 0.001) and pelvis (2.9 vs. 2.5, *p* = 0.004) (Table 1).

The median ISS was significantly higher in the LHM group (*p* = 0.002), where a greater proportion of cases had an ISS > 25 (82.9% vs. 69.0%, *p* = 0.001).

**Fig. 3:**
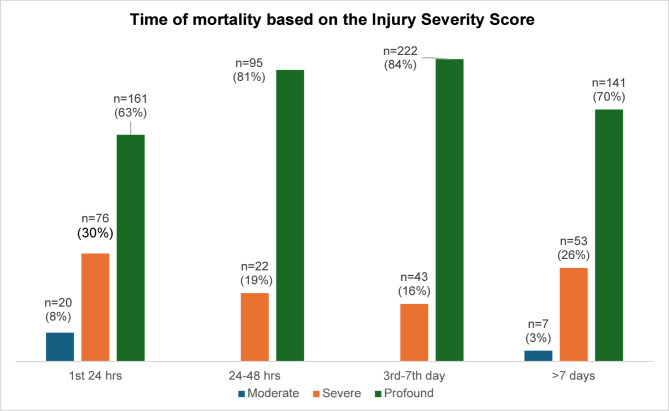
Time of in-hospital mortality and the Injury Severity Score (Moderate 9–15; severe 16–25, profound > 25)

As shown in Fig. 3, the time of death varied by ISS category. Within the first 24 h, 63% of deaths occurred in the “profound” injury group (ISS > 25), while 30% were in the “severe” injury group (ISS 16–25).

Procedural interventions also differed significantly between groups. The rates of exploratory laparotomy (41% vs. 22%, *p* = 0.001) and MTP activation (68.2% vs. 42.2%, *p* = 0.002) were significantly higher in the EHM group, indicating that hemorrhage is the leading cause of early death. In contrast, craniotomy/craniectomy was more common in the LHM group (26% vs. 4%, *p* = 0.001), consistent with traumatic brain injury as the primary cause of death. Additionally, the LHM group had higher complication rates, likely due to prolonged survival allowing complications to develop.

**Fig. 4:**
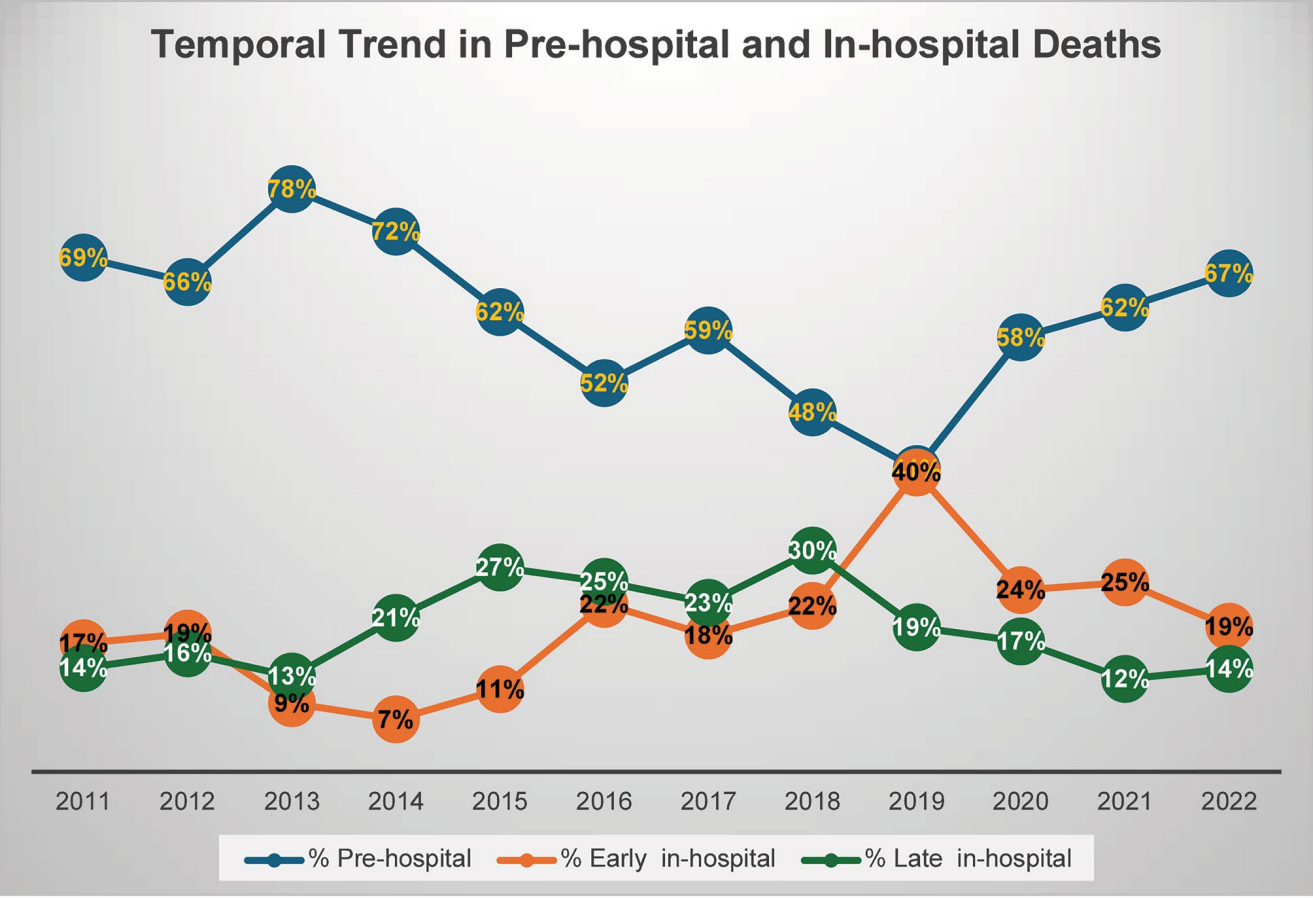
Temporal trend in pre-hospital and in-hospital mortality

Figure 4 shows the temporal trends in pre-hospital and in-hospital mortality over the study period. Pre-hospital deaths peaked in 2013 (78%, *n* = 153), followed by a gradual decline (R^²^ = 0.21), reaching its lowest point in 2019 (41%, *n* = 88). RHM fluctuated between 7% (*n* = 16, year 2014) and 40% (*n* = 87, year 2019), exhibiting an overall upward trend (R^²^ = 0.312). Notably, in 2019, pre-hospital and EHM rates were nearly identical (41% vs. 40% respectively). In contrast, LHM remained stable, ranging from 13% (*n* = 25 in 2013) to 30% (*n* = 48 in 2018), with no significant upward or downward trend. The overall trend reveals dynamic fluctuations between early and late in-hospital mortality. Early and late mortality rates were comparable in 2011, but after a three-year decline, early mortality rebounded and became similar to late mortality again. From 2019 onward, early mortality predominated, though the two rates became comparable again in 2022. This pattern indicates alternating periods of predominance between early and late in-hospital mortality, highlighting a cyclical trend over time.

### Age-based analysis

Table 2 shows the mechanism of injury, injury severity, comorbidities, and time of mortality among seven age groups. MVC was the primary cause of injury in patients aged 10–30, whereas falls were more common in those aged 31–40 and over 60 years. The highest ISS was observed in patients aged 21–30 and 51 − 50. Comorbidities, including diabetes, hypertension, and heart disease, were more prevalent in patients > 60 years. Death within 24 h was more common in patients aged 21–40, while death within 24–48 h occurred most frequently in the 0–10 age group. Mortality between 3 and 7 days was higher in patients aged 31–60, while death after 7 days was more frequent in patients > 60 years.

**Table 2:** Characteristics of mortalities based on age groups

	0–10 (*n* = 20)	11–20 (*n* = 71)	21–30 (*n* = 227)	31–40 (*n* = 146)	41–50 (*n* = 103)	51–60 (*n* = 74)	> 60 (*n* = 89)	*P*-value
**Mechanism of injury**								
MVC	4 (20.0%)	46 (64.8%)	92 (40.5%)	44 (30.1%)	27 (26.2%)	20 (27.0%)	11 (12.4%)	0.001 for all
VRU	9 (45.0%)	13 (18.3%)	72 (31.7%)	40 (27.4%)	34 (33.0%)	24 (32.4%)	22 (24.7%)	
Falls	5 (25.0%)	6 (8.5%)	45 (19.8%)	41 (28.1%)	25 (24.3%)	22 (29.7%)	51 (57.3%)	
Violence-related injury	1 (5.0%)	2 (2.8%)	5 (2.2%)	7 (4.8%)	4 (3.9%)	1 (1.4%)	0 (0.0%)	
Hit by objects	0 (0.0%)	1 (1.4%)	2 (0.9%)	4 (2.7%)	3 (2.9%)	5 (6.8%)	1 (1.1%)	
**Comorbidities** Diabetes mellitus % Hypertension % Cardiac diseases % Kidney diseases %	0 0 0 0	0 0 0 0	1.3 0.6 0.9 0	2.2 2.2 1.6 0	13.2 12.7 6.5 0	25.5 26.1 20.7 0	73.5 74.6 58.3 7.7	0.001
**ISS** median/IQR	28 (26–34)	30 (25–38)	33 (27–38)	30 (26–38)	30 (25–38)	34 (26–38)	26 (20–30)	0.001
**GCS at the ED** median /IQR	3 (3-5.5)	3 (3–3)	3 (3–3)	3 (3–3)	3 (3-12.5)	3 (3–8)	9 (3–15)	0.001
**Massive transfusion protocol activation**	0 (0.0%)	23 (65.7%)	60 (50.4%)	31 (41.9%)	22 (41.5%)	22 (64.7%)	5 (16.1%)	0.001
**Sepsis**	1 (7.1%)	5 (10.4%)	14 (8.8%)	6 (6.7%)	9 (12.9%)	9 (18.8%)	12 (25.5%)	0.020
**Hospital Mortality**								0.001 for all
Within 24 h	5 (25.0%)	17 (23.9%)	64 (28.2%)	44 (30.1%)	18 (17.5%)	14 (18.9%)	13 (14.6%)	
24–48 h	6 (30.0%)	9 (12.7%)	24 (10.6%)	16 (11.0%)	17 (16.5%)	7 (9.5%)	9 (10.1%)	
3–7 days	3 (15.0%)	24 (33.8%)	79 (34.8%)	62 (42.5%)	40 (38.8%)	29 (39.2%)	25 (28.1%)	
> 7 days	6 (30.0%)	21 (29.6%)	60 (26.4%)	24 (16.4%)	28 (27.2%)	24 (32.4%)	42 (47.2%)	

### Mechanism of injury and ISS-based analysis

**Fig. 5:**
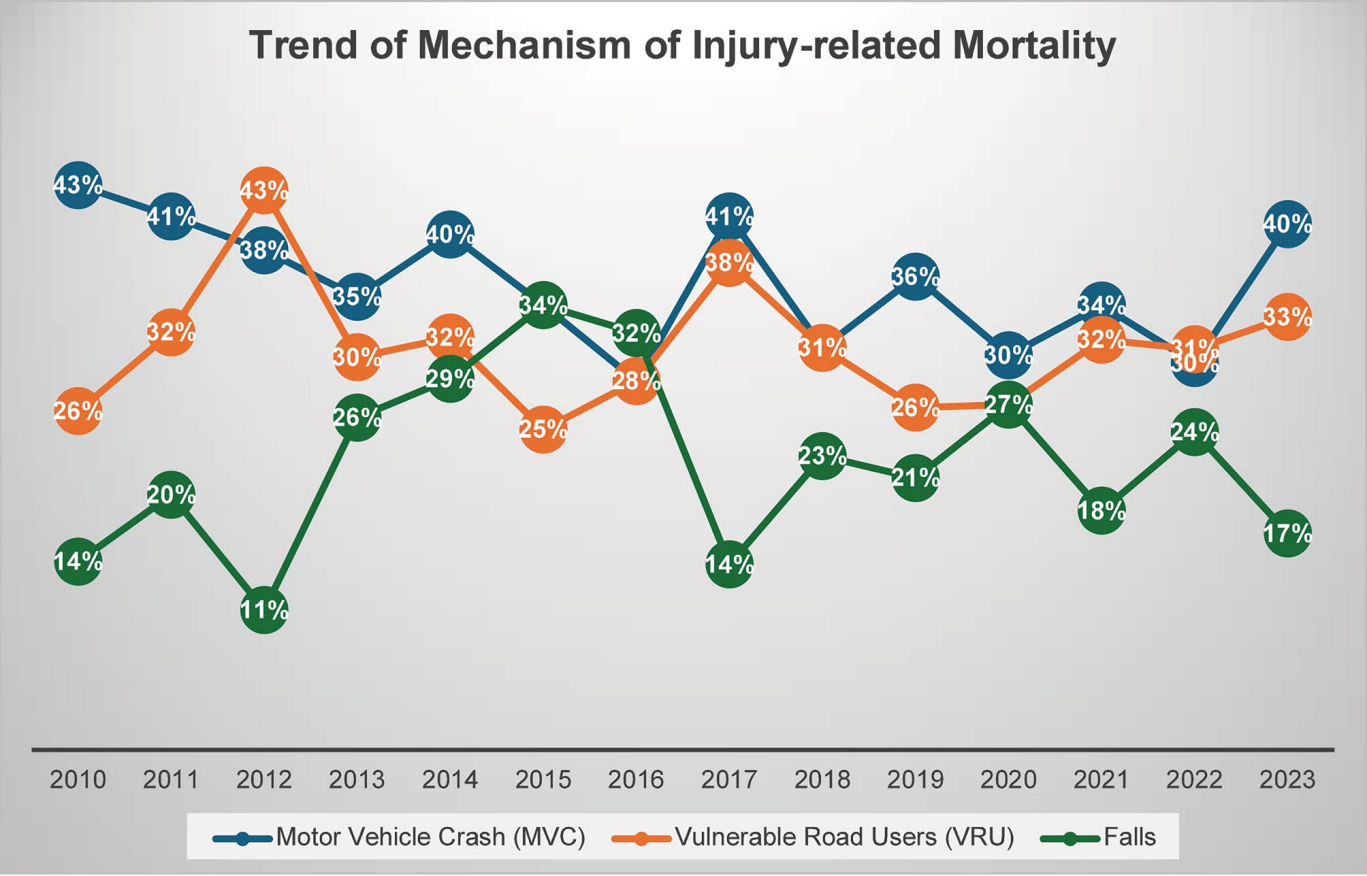
Trend of aggregated mortality by different mechanisms of injury

Figure 5 demonstrates trends in mortality related to mechanisms from 2010 to 2023, revealing dynamic shifts in causes of trauma-related deaths. For MVC-related mortality, the trend showed an upward trend in its contribution (R^2^ = 0.216). The proportion of MVC-related deaths ranged from 28 to 43%, with notable peaks in 2014 (40%) and 2017 (41%). Injuries among VRUs reached highs of 43% in 2012 and 38% in 2017 but remained relatively stable (R^²^ = 0.008). In contrast, fall-related deaths showed the highest variability (R² = 0.007), with low rates (14–26%) in earlier years, rising sharply in later years, peaking at 34% in 2015 and 27% in 2020. These patterns suggest that while MVCs and VRU injuries remained relatively stable, falls became a growing concern after 2013 before declining sharply in 2017. This may reflect a shift in the primary mechanisms of trauma-related mortality over the study period.

### Predictors of mortality

Multivariable analysis identified the admission systolic SI as an independent predictor of EHM (OR 2.23; 95% CI 1.09–4.52). In contrast, head injury (OR 7.14; 95% CI 2.44–20.00), pelvic injuries (OR 3.70; 95% CI 1.19–11.11), and sepsis (OR 6.25; 95% CI 1.22–33.33), predicted LHM (Table 3).

**Table 3:** Multivariable analysis for predictors of early in-hospital mortality

Variable	Odds ratio	95% confidence intervals		*P* value
Age in years	1.023	1.001	1.045	0.039
Glasgow Coma Scale at ED	0.911	0.826	1.005	0.063
Injury severity score	1.006	0.976	1.037	0.712
Sepsis	0.165	0.033	0.820	0.028
Systolic shock index	2.226	1.095	4.525	0.027
Head injury	0.138	0.046	0.415	< 0.001
Abdominal injury	1.178	0.534	2.599	0.684
Pelvic injury	0.270	0.086	0.844	0.024
Massive transfusion protocol activation	1.375	0.597	3.170	0.455

## Discussion

This observational study, which utilized data from a level 1 trauma center, analyzed 1,010 cases of in-hospital trauma-related mortality over 13 years. EHM occurred mainly among younger individuals compared to LHM. Vulnerable road users were more frequently associated with EHM, while falls from height were more common in LHM. Clinical characteristics also differed significantly, with early deaths being associated with worse shock indices and physiological instability, likely due to hemorrhage. In contrast, late deaths were more often linked to head injuries. Injury patterns also varied; while abdominal and pelvic injuries, both widely recognized causes of hemorrhage, were more prevalent in early deaths, head injuries contributed more significantly to late deaths. On multivariable analysis, sepsis and head and pelvic injury were predictors of late mortality, whereas systolic SI predicted early deaths. Most in-hospital deaths (40.9%) occurred within the first 24 h after arrival.

In our study, the overall incidence of trauma-related mortality in Qatar was 8.1 per 100,000 population. In contrast, a 6-year observational study from Abu Dhabi Emirate reported a lower overall incidence of 2.6 per 100,000 population. With Abu Dhabi, the Al-Ain region had the highest standardized mortality rate among hospitalized trauma patients (3.37 per 100,000 population), followed by Abu Dhabi City (2.48 per 100,000) and Al Dhafra (1.45 per 100,000) [20]. These disparities may be attributed to the variations in population demographics, duration of the study, quality of data collected, driving behaviors, construction activities, and occupational risks, all of which could significantly influence trauma-related mortality rates. Moreover, our population-based analysis of trauma-related mortality (2011–2022) revealed distinct temporal trends over the 13 years. The significant decline in mortality rate from 2011 to 2017 is likely attributed to improvements in trauma care protocols, stricter enforcement of seatbelt laws, and occupational safety campaigns [21]. However, this progress was reversed post-2017, with mortality rates rising sharply by 2022, a trend that correlates strongly with Qatar’s rapid infrastructure expansion in preparation for the FIFA World Cup 2022.

Furthermore, the COVID-19 pandemic introduced unique shifts in trauma epidemiology, with a marked overrepresentation of delivery motorcyclists among VRUs. This demographic shift likely contributed to the observed fluctuations in trauma-related mortality, underscoring the impact of socioeconomic changes on injury patterns. A comparative analysis of GCC nations reveals divergent trends in trauma mortality over the past decade. While countries like Qatar and the UAE demonstrated declines in mortality following investments in advanced trauma systems, data standardization, and preventive policies [22], others faced rising mortality rates driven by rapid urbanization, increasing road traffic incidents, and inadequately regulated occupational hazards associated with construction activities [23]. These disparities underscore the crucial role of policy enforcement and infrastructure development in preventing injuries. Moreover, earlier studies have suggested that reductions in trauma-related deaths during the early 2010s were primarily associated with improved emergency medical services (EMS) and stricter traffic regulations [13, 24, 25]. For instance, in the United Arab Emirates, the death rate due to road traffic injuries decreased substantially from 6.1 to 3.1 per 100,000 population by 2021 [26]. However, this progress has been partially offset in recent years, likely due to unchecked population growth, higher vehicle usage, and insufficient mitigation of construction-related injuries.

Fall-related injuries have emerged as a significant public health challenge in rapidly developing Middle Eastern countries, exacerbated by the unprecedented demand for construction. Our findings align with an earlier study from Qatar, which identified falls from height, being hit by heavy objects, and MVCs as the most common mechanisms of workplace-related traumatic injuries [27]. Supporting this, Consunji et al. [28] reported falls from height and being hit by heavy objects as the primary causes. Similarly, in the UAE, falls from heights, falling objects, and powered machinery were the most frequent mechanisms of occupational injuries [29]. These findings underscore the need for stricter enforcement of workplace safety regulations, enhanced training programs for workers, and targeted interventions to mitigate fall-related injuries in high-risk industries.

The present study also highlighted the persistently high burden of pre-hospital deaths (62%) in Qatar, indicating the severity of injuries sustained before hospital arrival. This finding aligns with a systematic literature review of 19 studies that reported pre-hospital death rates of 14.6–47.6%, with 4.9–11.3% deemed definitely preventable and 25.8–42.7% as potentially preventable [30]. Notably, Oliver et al. [31] study corroborates this, emphasizing that many pre-hospital deaths involve potentially survivable injuries, yet timely pre-hospital interventions are often inadequate. These observations suggest that while injury severity is a key factor, suboptimal pre-hospital care, such as prolonged response times, inadequate hemorrhage control, or lack of advanced life support, may contribute significantly to preventable mortality. Furthermore, our findings demonstrate a significant temporal shift in trauma-related mortality patterns, with pre-hospital deaths peaking in 2013 before declining to the lowest point in 2019. Notably, we have only the data on the frequency of pre-hospital deaths; detailed information regarding the specific causes of death is unavailable. However, this trend may be attributed to advancements in pre-hospital care, such as improved hemorrhage control, shorter transport times, critical care paramedic interventions (including intubation, medications, and CPR), and stricter enforcement of road safety regulations [32]. Moreover, in our cohort, early in-hospital mortality reflected an overall increasing trend. This increase could be associated with advancements in pre-hospital care, which implies successful ‘scoop-and-run’ strategies that enable the transportation of critically injured patients to the hospital for definitive care. This suggests that while pre-hospital advancements save lives, they also shift the burden of mortality to the early in-hospital phase, necessitating targeted interventions for hemorrhage control (e.g., pre-hospital blood products).

Prior research from our center indicated that 32% of trauma-related in-hospital deaths occurred early, within the first 48 h post-trauma [12]. In a multicenter study of 114,220 trauma patients, Sangji et al. [33] observed early deaths (≤ 48 h) in 39% of cases, highlighting variability across trauma center performance levels. In contrast, our findings reveal a significantly higher early mortality rate (53%) compared to these benchmarks, suggesting potential differences in injury severity, pre-hospital care efficiency, or institutional protocol.

Our analysis reveals that the clinical characteristics of trauma patients admitted to the hospital demonstrate significant differences between those with EHM and those with LHM. Early mortality was associated with severe physiological derangements upon admission, evidenced by significantly lower systolic and diastolic blood pressures and GCS, which are widely accepted as being caused by hemorrhage. Elevated shock indices in early deaths further underscore the potential role of hemorrhage or even devastating brain injuries in the early phase of trauma. These findings align with established literature implicating hemorrhage as the predominant driver of early deaths [34], which is further supported by the elevated shock indices. Several studies have identified head injuries and bleeding as the leading causes of immediate death in trauma patients [8, 10, 35, 36]. Meislin et al. [10] reported that neurological trauma and circulatory collapse (i.e., hemorrhage) were responsible for over 80% of early deaths. Sauaia et al. [36] reported that among trauma deaths occurring within 48 h of injury, exsanguination accounted for 51% of cases, primarily due to lacerations to the liver, heart, or major blood vessels. Extrapolating these findings to our cohort, approximately 51% of pre-hospital and early in-hospital deaths in Qatar (*n* ≈ 1000) may have resulted from exsanguination, and thus, most were potentially preventable. However, this remains speculation, as our study lacked post-mortem data to confirm the exact causes of death.

A previous study from our center reported that late mortality is frequently linked to complications arising from initial injuries, particularly multiorgan failure (MOF) and sepsis [13]. Similarly, Sauaia et al. [36] reported that among patients who died more than one week post-injury, 61% succumbed to MOF. The immune response triggered by the primary injury increases susceptibility to secondary infections, thereby elevating the risk of sepsis and related complications, such as acute respiratory distress syndrome (ARDS) and multiple organ failure (MOF). Notably, mortality rates among septic trauma patients range between 19.5% and 23% [37]. Also, the predominance of head injuries in late deaths implies that secondary brain injury mechanisms contribute to delayed mortality.

Our analysis reveals evolving epidemiological patterns in trauma mechanisms: while MVCs and pedestrian injuries maintained stable incidence rates, fall-related trauma surged post-2018. This paradigm shift reflects an aging population that is increasingly vulnerable to low-energy falls, as well as declining occupational falls among younger workers, due to improved workplace safety regulations. Pedestrian injuries, often involving high-energy impacts, tend to result in complex injuries and immediate or earlier death, while falls from a height might result in multiple, less severe, injuries that are initially survivable but lead to longer hospital stays that allow time for complications to develop over time [38–40]. For the prevention of MVCs and pedestrian injuries, enhanced speed enforcement, redesign of pedestrian-protective infrastructure, and targeted public awareness campaigns are necessary. Additionally, the implementation of geriatric mobility programs and the strengthening of occupational safety systems are required for fall prevention. These interventions should be guided by real-time trauma registry data and implemented through multidisciplinary collaboration between trauma systems, urban planners, and occupational health authorities.

## Limitations

Our study has several limitations. First, as a retrospective, single-center study, it is subject to potential biases that could affect the generalizability of the study findings. Second, the study presents a gender disparity, as most patients were males, which reflects the unique gender distribution of the region. Third, the study did not capture the number of falls from heights (> 2 m) or while standing, as these indicate two different populations. Fourth, the outcomes primarily focused on in-hospital mortality, lacking crucial data on pre-hospital deaths and post-discharge outcomes. Finally, detailed information on the direct causes of trauma-related fatalities was unavailable due to a lack of post-mortem examination, which is not routinely performed due to cultural considerations. Therefore, pre-hospital in-depth analysis was not feasible.

## Conclusion

This study provides crucial insights into trauma-related mortality patterns in Qatar, revealing distinct clinical and temporal trends. Early mortality is prevalent among vulnerable road users and is strongly linked to hemorrhage and hemodynamic instability, as evidenced by elevated shock indices. Late mortality, on the other hand, is associated with severe head injuries and polytrauma. Our findings underscore the dynamic nature of trauma mortality, with MVCs and falls remaining persistent contributors. These findings underscore the need for targeted interventions, including optimized pre-hospital hemorrhage control, in-hospital shock index monitoring, and enhanced neurotrauma management protocols. Furthermore, there is a need to strengthen injury prevention through stricter road safety laws and public awareness campaigns for high-risk groups. Implementation of these evidence-based approaches could significantly reduce preventable trauma deaths, advance Qatar’s trauma care system, and provide a model for regional collaboration. Future research should evaluate the effectiveness of interventions and long-term patient outcomes to refine trauma care strategies further.

## Data Availability

All data were given in the manuscript, tables and figures. Data can be obtained from the PI after reasonable request and signing data sharing agreement and approval from the medical research center at Hamad Medical Corporation, Doha, Qarar.
